# Control of Foodborne *Staphylococcus aureus* by Shikonin, a Natural Extract

**DOI:** 10.3390/foods10122954

**Published:** 2021-12-01

**Authors:** Yangli Wan, Xiaowen Wang, Pengfei Zhang, Meng Zhang, Mingying Kou, Chao Shi, Xiaoli Peng, Xin Wang

**Affiliations:** College of Food Science and Engineering, Northwest A & F University, Xianyang 712100, China; wanyangli@nwsuaf.edu.cn (Y.W.); 2020051067@nwafu.edu.cn (X.W.); ZMXN540395587@nwafu.edu.cn (P.Z.); 2019051991@nwafu.edu.cn (M.Z.); 2019055772@nwafu.edu.cn (M.K.); meilixinong@nwsuaf.edu.cn (C.S.); pxlpxh@163.com (X.P.)

**Keywords:** *S. aureus*, shikonin, antibacterial activity, SEA, Hla, biofilm

## Abstract

Foodborne *Staphylococcus aureus* (*S. aureus*) has attracted widespread attention due to its foodborne infection and food poisoning in human. Shikonin exhibits antibacterial activity against a variety of microorganisms, but there are few studies on its antibacterial activity against *S. aureus*. This study aims to explore the antibacterial activity and mechanism of shikonin against foodborne *S. aureus*. The results show that the minimum inhibitory concentrations (MICs) and the minimum bactericidal concentrations (MBCs) of shikonin were equal for all tested strains ranging from 35 μg/mL to 70 μg/mL. Shikonin inhibited the growth of *S. aureus* by reducing intracellular ATP concentrations, hyperpolarizing cell membrane, destroying the integrity of cell membrane, and changing cell morphology. At the non-inhibitory concentrations (NICs), shikonin significantly inhibited biofilm formation of *S. aureus,* which was attributed to inhibiting the expression of *cidA* and *sarA* genes. Moreover, shikonin also markedly inhibited the transcription and expression of virulence genes (*sea* and *hla*) in *S. aureus*. In addition, shikonin has exhibited antibacterial ability against both planktonic and biofilm forms of *S. aureus*. Importantly, in vivo results show that shikonin has excellent biocompatibility. Moreover, both the heat stability of shikonin and the antimicrobial activity of shikonin against *S. aureus* were excellent in food. Our findings suggest that shikonin are promising for use as a natural food additive, and it also has great potential in effectively controlling the contamination of *S. aureus* in food and reducing the number of illnesses associated with *S. aureus*.

## 1. Introduction

*Staphylococcus aureus* is an important foodborne pathogen, causing human food poisoning and infections worldwide [1]. It produces a wide variety of toxins, but enterotoxins (SEs) and hemolysins are known to be the main pathogenic factors of human food poisoning and infections, respectively [1,2]. Currently, 29 types of SEs have been reported, including the classical SEs (SEA to SEE) and the novel SEs (SEG to SEl27) [3,4]. The most common SEs associated with food poisoning is SEA, which is responsible for approximately 80% of food poisoning outbreaks [4,5]. In addition, hemolysins can bind to most of the cells and tissues in the host, damage and lyse their cell membrane structures, and has strong pathogenic power. Currently, hemolysins can be divided into four types according to their genetic determinants: α, β, γ and δ. Among these hemolysins, α-hemolysin (Hla) is the main virulence factor of *S. aureus* for human infections [2].

In addition to SEs and hemolysin, another key factor of foodborne *S. aureus* causing food poisoning is biofilm. Biofilm formation represents a protected mode of growth that allows cells to survive in hostile environments and also disperses to colonize new niches [6]. Biofilms are essential to protect bacteria from various stress conditions, including antibacterial and antifouling agents, shear force, host phagocytosis and free radical action. In addition, the microcolonies in the biofilm mediate the propagation process through dissociation. The dissociated microcolonies migrate from the original biofilm colony to the uninfected area of the host, attaching and promoting the formation of new biofilm [7]. The biofilm formed by harmful bacteria during food processing can increase microbial residues. Therefore, the inability to strictly clean and disinfect the processing equipment will not only damage the equipment, but also become a serious potential source of pollution, thereby contaminating food and spreading foodborne diseases.

Shikonin is a monomeric component extracted from comfrey roots, which has been traditionally used as a cure for burns, inflammations, and wounds and as a dye for staining fabrics, food colorants, and food antioxidants in many countries [8,9]. Although the safety of shikonin, which is considered to be a food colorant and food antioxidant, has not been systematically evaluated, several in vivo studies have confirmed that shikonin is safe [10,11]. Recently, pre-clinical animal experiments and in vitro cell experiments have confirmed that shikonin has a wide spectrum of biological functions, such as anti-tumor, anti-inflammatory and wound healing [12,13,14]. In addition, shikonin has shown antibacterial, antifungal, and antiviral activity [15,16]. Moreover, shikonin has been shown to be especially active against Gram-positive bacteria including *S. aureus* [15]. However, its antibacterial activity against foodborne *S. aureus* has not been well documented.

In short, this study aims to explore the antibacterial activity of shikonin against foodborne *S. aureus*. First, the minimum inhibitory concentrations (MICs), the minimum bactericidal concentrations (MBCs), growth curve, and the non-inhibitory concentrations (NICs) of shikonin against *S. aureus* was determined to assess its bacteriostatic activity. The bactericidal mechanism of shikonin for *S. aureus* was determined by measuring the changes in cell membrane potential, intracellular ATP concentration, cell membrane integrity and cell morphology. In addition, the effect of the non-inhibitory concentrations of shikonin on the formation of biofilm and the expression of virulence factors of *S. aureus* was determined by hemolysin assays, Western blot, and reverse transcription quantitative real-time polymerase chain reaction (qRT-PCR), and microtiter plate assay. The clearance rate of shikonin on *S. aureus* biofilm was detected by colony count. Finally, the heat stability of shikonin, the safety assessment of shikonin in mice, and the antimicrobial activity of shikonin against *S. aureus* in beef, seaweed and mulberry juice samples were assessed.

## 2. Materials and Methods

### 2.1. Reagents

Tryptic Soy Agar (TSA) and Tryptic Soy Broth (TSB) were purchased from Qingdao Hope Bio-Technology Co, Ltd. (Qingdao, China); Shikonin (HPLC ≥ 98%, CAS 517-89-5) was purchased from Shanghai Yuanye Biotechnology Co., Ltd. (Shanghai, China) and the chemical structure of shikonin is shown in Figure S1 Dimethyl sulfoxide (DMSO), Monoclonal anti-SEA antibody (Cat. No. S7656) and DiBAC_4_(3) fluorescent probe were purchased from Sigma-Aldrich; ATP detection kit (Cat. No. S0026) was purchased from Biyuntian Biotechnology Co., Ltd. (Shanghai, China); Live/Dead^®^ BaclightTM bacterial activity detection kit was purchased from Thermo Fisher Scientific (Waltham, MA, USA); Monoclonal anti-Hla antibody (Cat. No. ab52922) was purchased from Abcam (Cambridge, UK). Seaweed, beef and mulberry juice were purchased from the supermarket in Yangling City, Shaanxi Province.

### 2.2. Strains

*S. aureus* ATCC 29213, ATCC 25,923 and seven foodborne *S. aureus* strains (A86, A48, 13#, S2, S4, 124, 265) were used in this study. To explore the antibacterial mechanism of shikonin, follow-up experiments were carried out with a standard strain ATCC29213 that has been fully recognized for its phenotype and genotype and has a strong ability to form biofilms [17].

To prepare *S. aureus* working suspensions as previously described [18], all *S. aureus* strains from frozen stock cultures were streaked onto TSA and incubated at 37 °C for 24 h. Then, one single colony was inoculated into TSB and incubated at 37 °C for 16 h. After centrifugation at 8000× *g* at 4 °C for 5 min, bacteria cells were washed three times with sterile phosphate buffered saline (PBS) and resuspended in sterile TSB to achieve 10^6^ CFU/mL as working bacterial suspensions.

### 2.3. MICs and MBCs of Shikonin against S. aureus

The MICs of shikonin against planktonic *S. aureus* were determined using broth microdilution assays as previously described [18]. The MICs of shikonin was assessed as the lowest concentration that inhibited 99% of *S. aureus* compared to the untreated control (only 1% of DMSO).

To determine the MBCs, 100 μL of each culture from the MICs wells and all other wells with no visible growth was spread on the TSA plate as previously described [18]. After the plate was cultured at 37 °C for 24 h, MBCs was defined as the lowest concentration of shikonin that reduced the CFU of the initial bacterial inoculum by 99.9%.

### 2.4. NICs of Shikonin against S. aureus

The concentration that does not affect the growth of *S. aureus* is defined as the NICs of shikonin. The NICs of shikonin on *S. aureus* were determined as described by Shi et al. (2017) with some modifications [19]. An amount of 125 μL of prepared bacterial suspension was added to the honeycomb plate (use with automatic growth curve tester), and then, 125 μL of shikonin solutions were added to achieve the final mass concentrations of 0, 1/64 MIC, 1/32 MIC, 1/16 MIC, 1/8 MIC, 1/4 MIC, 1/2 MIC, MIC and 2 MIC. The samples were placed in an automatic microbial growth curve analyzer (Labsystems, Helsinki, Finland), and incubated at 37 °C for 24 h, and then the optical density of each sample at 600 nm wavelength was measured at 1 h intervals. Finally, the growth curve was drawn according to the measured optical density.

### 2.5. The Effect of Shikonin on the Membrane Potential of S. aureus

The cell membrane potential was measured as described previously [18]. Briefly, the prepared bacterial suspension was added to the black ELISA plate and incubated at 37 °C for 30 min. Then, the shikonin was added to the sample wells to achieve final mass concentrations of 0, MIC, and 2 MIC. After the ELISA plate was incubated at 37 °C for 1 h, DiBAC_4_(3) fluorescent probe dye was added to each well to achieve a final concentration of 1 μM and incubated for 10 min at room temperature in the dark. The fluorescence intensity of each well was detected using a fluorescence microplate reader (Spectra Max M2; Molecular Devices, San Jose, CA, USA) at 492 nm excitation and 515 nm emission wavelengths. Finally, the change in membrane potential was obtained by comparing the fluorescence intensity.

### 2.6. The Effect of Shikonin on the Intracellular ATP Concentration of S. aureus

The method for measuring intracellular ATP concentration was slightly modified as described previously [18]. Briefly, 1 mL of the prepared bacterial suspension was mixed with different mass concentrations of shikonin solutions to achieve final mass concentrations of 0, MIC and 2 MIC and incubated at 37 °C for 30 min. The samples were then placed on ice and lysed by ultrasound. The lysed cells were used to detect the intracellular ATP concentration of *S. aureus*. After the samples were centrifuged for 5 min at a speed of 5000× *g*, the ATP concentration of each sample supernatant was detected and calculated using Biotechnology ATP detection kit (Cat. No. S0026).

### 2.7. The Effect of Shikonin on the Membrane Integrity of S. aureus

The cell membrane integrity was performed as described previously [20]. Briefly, the activated bacteria solution was treated with 0.85% NaCl and 70% isopropanol solution for 1 h, respectively, to obtain live and dead bacteria. The live bacteria group and the dead bacteria group were mixed in different proportions to prepare different ratios of live bacteria (0%, 10%, 50%, 90%, and 100%) to establish a standard curve according to the instructions of the Live/Dead^®^Baclight^TM^ kit (Cat. No. L7012).

Shikonin was added to the viable cell suspension to achieve the final mass concentrations of 0, MIC and 2 MIC, and incubated at 37 °C for 30 min. Subsequently, the bacterial suspensions were centrifuged at 10,000× *g* for 3 min. An amount of 200 μL of cell suspension was added to a black 96-well plate (Thermo Fisher, Waltham, MA, USA), and then an equal volume of mixed SYTO9/PI dye was added and incubated at room temperature for 10 min in the dark. The fluorescence intensity was measured with a multifunctional microplate reader (spark, Tecan Austria Gmbh) using excitation wavelength (SYTO9 for 480 nm and PI for 490 nm) and an emission wavelength (SYTO9 for 500 nm and PI for 635 nm). Finally, the percentage of viable cells in each sample was calculated based on the established standard curve.

### 2.8. The Effect of Shikonin on the Cell Morphology of S. aureus

The morphology of *S. aureus* was observed by using a scanning electron microscope as described previously [18]. After the bacterial cells were treated with shikonin (0, MIC, 2 MIC) at 37 °C for 1 h, all samples were centrifuged at 10,000× *g* for 10 min and washed three times with PBS. Subsequently, the bacterial cells were first fixed in 2.5% glutaraldehyde solution at 4 °C for 10 h, and then placed in 1% acidic acid for post-fixation at 4 °C for 6 h. After being fixed, the bacterial cells were dehydrated gradually with 30%, 50%, 70%, 80%, 90% and 100% alcohol at room temperature for 20 min. Finally, the bacteria cells surface was sprayed with gold, and placed in field emission electron microscope (S-4800; Hitachi, Tokyo, Japan) for observation and photographing.

### 2.9. The Effect of Shikonin on the Biofilm of S. aureus

The biofilm biomass was carried as described previously by J. H. Li et al. (2021) [21]. Shikonin was added to the prepared bacterial suspension in 96-well plates to make the final concentration reach 0, 1/64 MIC, 1/32 MIC, 1/16 MIC (at the same time, the shikonin solution of different concentrations without bacterial suspension were used as background blank control). After incubation at 37 °C for 24 h, all samples were measured for optical density at 630 nm. The supernatant was removed and gently washed three times with distilled water. After air drying, each well was stained with 200 μL of 1% crystal violet for 20 min. Subsequently, crystal violet was removed and gently washed twice times with sterile distilled water. After air drying, each well was stained with 200 μL 33% glacial acetic acid for 20 min. Finally, the optical density was measured at 570 nm. The biofilm biomass of each well was expressed as OD_570 nm_/OD_630 nm_.

Among them, OD_570 nm_ and OD_630 nm_ comply with the following formula: OD_570 nm_ = OD_570_(treated) − OD_570_(blank), OD_630 nm_ = OD_630_(treated) − OD_630_(blank).

The biomass of the biofilm was visually observed under an optical microscope. As with the biofilm staining method mentioned above, all samples were stained with 1% crystal violet for 20 min, and then washed three times with distilled water. After the sample was dried, the biofilm was observed under an optical microscope (400× magnification).

### 2.10. RT-qPCR of S. aureus Biofilm Formation Key Genes (icaA, cidA, agrA, sarA) and Virulence Genes (Hla, Sea)

RT-qPCR of *icaA*, *cidA*, *agrA*, *sarA hla*, and *sea* genes were performed with minor modifications as described previously [17,21]. All the primers used in the study are listed in Table 1. Briefly, the bacterial suspension was treated with the non-inhibitory concentration of shikonin and 0.1% DMSO (negative control). RT-qPCR was performed using a 20 µL reaction mixture according to the SYBR Green Premix Ex Taq II kit (Takara, Kusatsu City, Japan) instructions. RT-qPCR reaction conditions: pre-denaturation at 95 °C for 30 s, 40 cycles of denaturation at 95 °C for 5 s, and extension at 56 °C for 40 s. The transcription level of the 16S rRNA gene was used as an internal reference, and the transcription levels of different genes in different samples were calculated according to the 2^−ΔΔCt^ method.

### 2.11. Colony Count

The colony count of *S. aureus* was performed with some slight modifications according to previous studies [22,23]. Briefly, 200 µL of bacterial suspension was added to the 96-well plate and placed at 37 °C for 24 h to pre-form biofilm. All wells were rinsed three times with PBS to remove planktonic cells. Subsequently, the biofilm was exposed to shikonin (0, MIC, 2 MIC, 10 MIC) and incubated at 37 °C for 1 h, 2 h, and 24 h, respectively. Afterward, all samples were washed three times with PBS and resuspended with 200 µL PBS. After 10-fold dilutions of each treated sample, 100 µL bacterial suspension was applied to TSA and incubated at 37 °C for 24 h.

### 2.12. Hemolytic Ability

The hemolytic ability was performed using sheep erythrocytes as described previously [24]. The *S. aureus* strains were cultured with 0, 1/64 MIC, 1/32 MIC and 1/16 MIC shikonin for 24 h. The supernatant was collected by centrifugation at 12,000× *g* for 10 min. The supernatants were filtered with a 0.22 μM filter membrane. Subsequently, 250 μL of the filtered supernatants were added to 1 mL of 6% sheep erythrocytes and mixed gently by inversion, and then incubated at 37 °C for 2 h. The supernatant was collected by centrifugation at 3800× *g* for 10 min. Finally, the optical density of the supernatant was measured at 450 nm. Positive (0.1% Trition X-100), negative TSB) were set. The percentage of hemolysis was determined based on the ratio of the sample to the positive control.

### 2.13. Western Blotting

SEA and Hla were determined by Western blot analysis as described previously [25], with some modifications. Bacteria cells and supernatants were collected after being treated with non-inhibitory concentration for 24 h. The bacterial cells were sonicated to obtain the whole cell lysate proteins, and the supernatant was filtered to obtain extracellular proteins. Before loading the sample, the protein concentration was determined using a micro-spectrophotometer (K5800C, KAIAO Technology, Beijing, China). The denatured samples were separated by SDS-polyacrylamide (12%) electrophoresis and transferred to 0.22 μM PVDF membrane. Then, the PVDF membrane was incubated with 5% skimmed milk at room temperature for 4 h, and incubated at 4 °C overnight for the primary antibody (Hla and SEA), while the secondary antibody was incubated at room temperature for 1 h. The PVDF membrane was evenly dropped with a chemiluminescence enhancing solution, and then exposed and imaged with chemiluminescence imager (Tanon, Shanghai, China).

### 2.14. Thermal Stability and Safety Assessment of Shikonin

Thermal stability: after shikonin was treated at 100 °C for 1, 3, 5 min or at 121 °C for 30 s, the changes in MIC and MBC of shikonin against *S. aureus* were detected as described in Section 2.3.

Animal experiments were approved by the Animal Protection and Utilization Committee of Northwest Agriculture and Forestry University and were conducted following the guidelines of the Animal Protection and National Institutes of Health. Thirty-day subacute toxicity experiments of shikonin in Kunming mice were performed as described previously [11]. Briefly, ten male Kunming mice, 5 weeks old, were purchased from the Experimental Animal Center of Xi’an Jiaotong University. After 1 week of adaptation, the mice were randomly assigned into two groups (each group *n* = 5). One group was intragastrically administered with shikonin at 800 mg/kg per day as the experimental group, and the other group was intragastrically administered with PBS containing 1% DMSO per day as the control group. The solvent or shikonin was gavaged orally once a day for up to 30 days. The mice were anesthetized with sodium pentobarbital and euthanized on days 30. The heart, liver, spleen, lung, kidney and small intestine were quickly dissected and fixed with 4% paraformaldehyde overnight. Each tissue was embedded in paraffin, sliced and stained with hematoxylin-eosin (HE). Finally, the morphology and structure of the tissue section were observed with a microscope, determining whether there were obvious lesions, and the images were collected and analyzed.

### 2.15. Application of Shikonin in Food Model

An amount of 5 g of beef and seaweed were weighed separately, and then soaked with 35 μg/mL shikonin solution for 5 min, so that shikonin was evenly distributed on each part of the surface of the beef and seaweed samples. In addition, shikonin was added to 5 mL of mulberry juice to make the final concentration 35 μg/mL. Then, a sensory evaluation of the three foods was executed. Three food models were added to the bacterial suspension to make a concentration of about 10^5^ CFU/mL or 10^5^ CFU/g. Positive (shikonin + *S. aureus*), negative (*S. aureus*) were set. After the different samples were treated at 25 °C for 24 h, the total number of colonies in the samples was calculated according to the GB 4789.2-2016 National food safety standard-Food microbiological examination-Aerobic plate count [26], at the same time, the sensory evaluation of the three foods was also evaluated. An amount of 5 g (or 5 mL) of the test sample was cut into pieces, placed in a sterilized mortar containing 45 mL of sterilized physiological saline, and fully ground to make a uniform dilution of 1:10. Subsequently, successive 10-fold gradient dilutions were performed, and the appropriate dilutions were selected for plate coating counting. Three replicates were set for each sample, and three plates were coated for each dilution.

### 2.16. Statistical Analysis

All experiments were repeated more than three times independently, and the data shown are the mean ± standard deviation. All experimental data were analyzed using the SPSS version 20.0 (SPSS Inc., Chicago, IL, USA). The significant difference between the two groups of data was performed by a Student’s T test. The significant differences between the three groups and more than three groups of data were performed by one-way analysis of variance. Levels of significance were indicated as follows: * *p* < 0.05; ** *p* < 0.01.

## 3. Result

### 3.1. MICs and MBCs

Shikonin has antibacterial activity against the 9 SEA-producing *S. aureus* strains shown in Table 2. The MIC of shikonin against strains 124, 265, S2, S4, 13# was 70 μg/mL; while the MIC of shikonin against ATCC29213, ATCC25923, A48, A86 strains was 35 μg/mL, indicating the latter four strains were more sensitive to shikonin. Moreover, the measurement results of the MBCs indicate that the MBC of shikonin for all strains was equal to MIC.

### 3.2. Growth Curve and NICs

**Figure 1:**
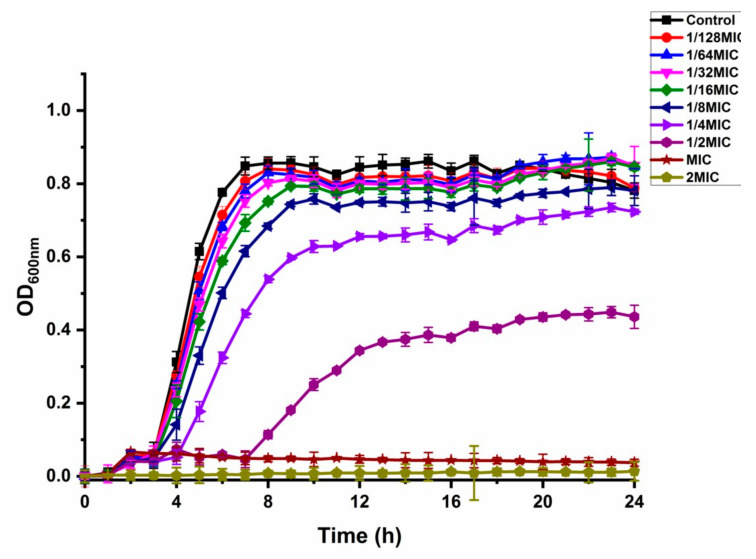
The effect of shikonin on the growth curve of strain ATCC29213. Each value was represented by the average of three independent experiments, and the line represents the standard deviation.

The effect of shikonin on the growth curve of the standard strain of *S. aureus* ATCC29213 was shown in Figure 1. The growth of strain ATCC29213 was totally inhibited by shikonin at MIC and 2 MIC. The growth curve of strain ATCC29213 had almost no effect by shikonin at 1/16 MIC, 1/32 MIC and 1/64 MIC. The concentrations of 1/16 MIC, 1/32 MIC and 1/64 MIC were determined as the NICs.

### 3.3. The Intracellular ATP Concentration of S. aureus

**Figure 2:**
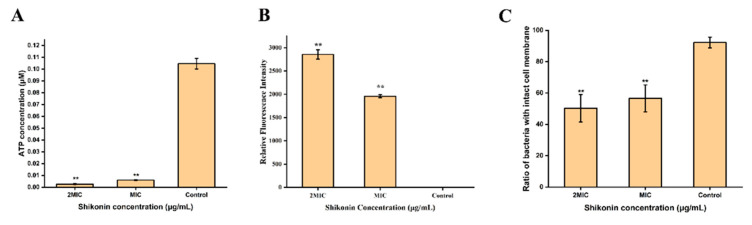
The effect of shikonin on the intracellular ATP concentration (**A**), cell membrane potential (**B**), cell membrane integrity (**C**) of strain ATCC29213. The average and standard deviation of three independent experiments were presented in a histogram. ** *p* < 0.01.

The standard curve was established according to the instructions of the intracellular ATP concentration detection kit (Figure S2). As shown in Figure 2A, the intracellular ATP concentration of strain ATCC29213 was detected at 0.1017 μmol/L in the untreated group, 0.0063 μmol/L in the MIC group, and 0.0023 μmol/L in the 2 MIC group. Compared with untreated group, the intracellular ATP concentration of shikonin-treated strain ATCC29213 was significantly decreased (*p* < 0.01).

### 3.4. The Cell Membrane Potential of S. aureus

The membrane potential of *S. aureus* ATCC29213 cells changed significantly after being treated with shikonin (Figure 2B). Compared with control group, the intracellular fluorescence intensity of shikonin-treated strain ATCC29213 increased significantly (*p* < 0.01), indicating that strain ATCC29213 was depolarized. The depolarization of strain ATCC29213 with shikonin at 2 MIC was more obvious than when the concentration was MIC.

### 3.5. The Integrity of S. aureus Cell Membrane

**Figure 3:**
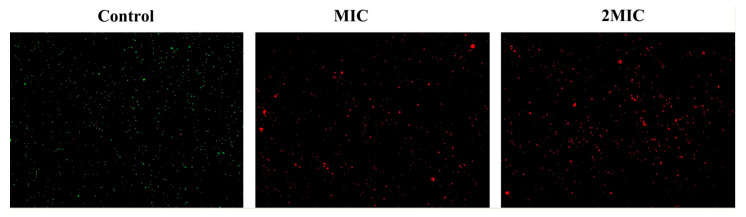
Observation of the effect of shikonin on the cell membrane integrity of strain ATCC29213 by laser confocal microscope (100× magnification).

According to the instructions of the Live/Dead^®^ BaclightTM Bacterial Activity Detection Kit, a linear relationship between the percentage of viable cells and the intensity of green fluorescence was established (Figure S3). As shown in Figure 2C, shikonin treatment reduced the percentage of viable cells of ATCC29213 significantly (*p* < 0.01). Similarly, the results of fluorescence microscopy show that shikonin treatment reduced the intensity of green fluorescence in the strain (Figure 3). On the contrary, shikonin increased the intensity of red fluorescence in the strain (Figure 3).

### 3.6. The Cell Morphology of S. aureus

**Figure 4:**
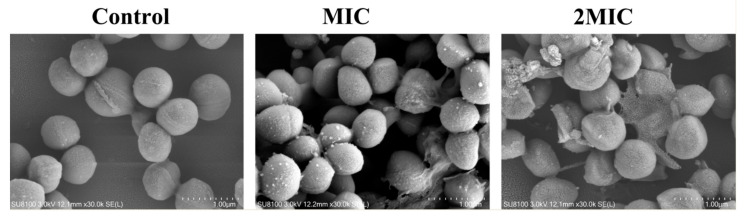
Scanning electron microscope observation of the effect of shikonin on the cell morphology of strain ATCC29213.

The morphological changes of ATCC29213 strain were observed by SEM under different concentrations of shikonin samples (Figure 4). The untreated ATCC29213 strain had a smooth and complete spherical shape, whereas the bacteria incubated with MIC of shikonin show a rough and vague boundary. As the concentration of shikonin increased, the cell morphology of strain ATCC29213 was more seriously damaged, and the number of damaged cells was greater.

### 3.7. The Biofilm Formation of S. aureus

**Figure 5:**
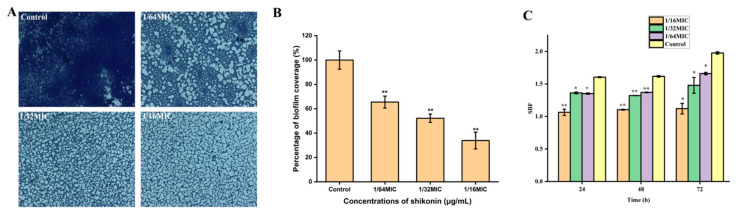
The effect of shikonin on biofilm formation of strain ATCC29213. (**A**) Observation of biofilm stained by crystals under optical microscope (400× magnification); (**B**) The biofilm coverage rate was quantified with the software ImageJ; (**C**) The effect of different shikonin treatment time on the formation of biofilm. ** *p* < 0.01, * *p* < 0.05.

After *S. aureus* strain ATCC29213 was treated with non-inhibitory concentrations of shikonin for 24 h, the formation of biofilm was quantified by crystal violet staining combined with optical microscope observation. The biofilm of the control strain was intact, while the biofilm formation was reduced when the strain was treated with shikonin (Figure 5A). With the increase of shikonin concentration, the formation of biofilm showed a downward trend (Figure 5B). Subsequently, the shikonin treatment time was extended to 48 h and 72 h, and compared with the 24 h treatment group, similar results were obtained (Figure 5C). In short, shikonin significantly inhibited the biofilm formation of *S. aureus* in a dose-dependent manner.

### 3.8. Shikonin Inhibited the Transcription of Cida and sarA Genes

**Figure 6:**
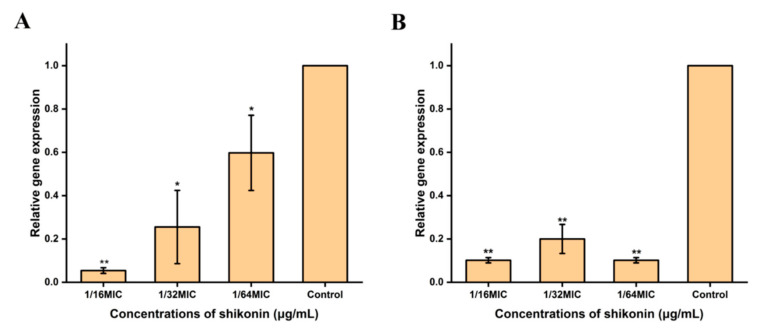
Shikonin inhibited the gene transcription of *cidA* and *sarA*. (**A**) The transcription of *cidA* of strain ATCC29213 treated with shikonin was detected by RT-qPCR; (**B**) The transcription of *sarA* of strain ATCC29213 treated with shikonin was detected by RT-qPCR. ** *p* < 0.01, * *p* < 0.05.

As shown in Figure 6, when *S. aureus* strain ATCC29213 was treated with different concentrations of shikonin, the gene transcription of *cidA* and *sarA* related to biofilm formation was inhibited, but there was no significant effect (*p* > 0.05) on the gene transcription of *agrA* and *icaA* (Figure S4).

### 3.9. Antibacterial Activity of Shikonin on S. aureus Bacteria in Biofilm

**Figure 7:**
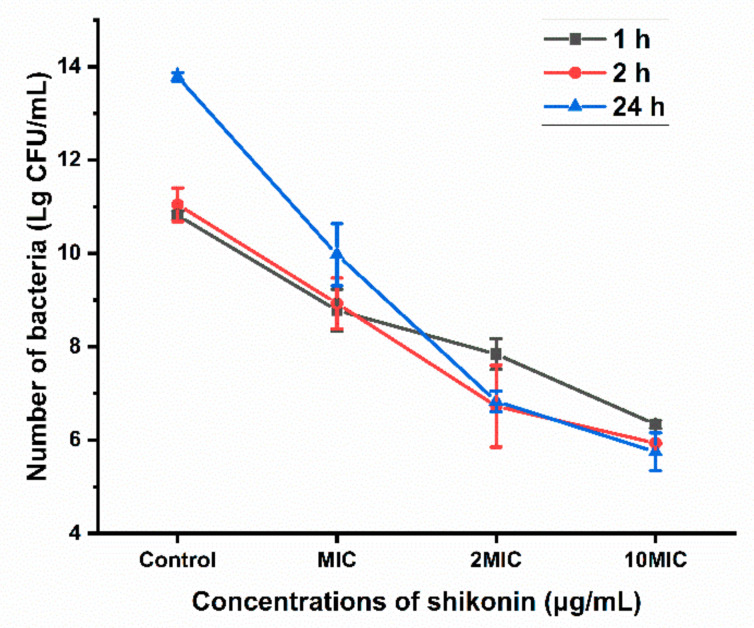
Shikonin inhibited the growth of *S. aureus* in the biofilm.

To determine the removal efficiency of shikonin on the biofilm, the colonies in the biofilm exposed to different concentrations of shikonin (0, MIC, 2 MIC, 10 MIC) were counted. As shown in Figure 7, shikonin inhibited the growth of *S. aureus* in the biofilm in a dose-dependent manner, and the inhibition of shikonin would be stronger when the treatment time was longer with the same concentration.

### 3.10. Shikonin Inhibited the Hemolytic Ability of S. aureus

**Figure 8:**
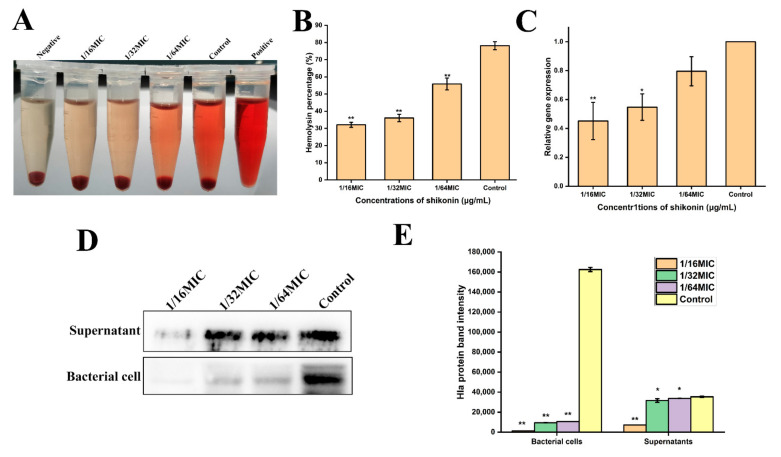
Shikonin at non-inhibitory concentration inhibited the hemolytic ability of *S. aureus*. (**A**,**B**) Changes in hemolytic ability of *S. aureus* strain ATCC29213 following treatment of shikonin; (**C**) The gene transcription of *hla*; (**D**,**E**) The protein expression of Hla was detected by Western blot and densitometry. ** *p* < 0.01, * *p* < 0.05.

As shown in Figure 8A,B, treatment with non-inhibitory concentrations of shikonin significantly inhibited the hemolytic ability of strain ATCC29213 (*p* < 0.01), and the degree of inhibition of hemolytic ability increased as the concentration increased. The results of RT-qPCR detection of *hla* gene transcription are shown in Figure 8C, indicating that shikonin treatment inhibited *hla* gene transcription. Meanwhile, Western blot results show that the expression of protein Hla was inhibited in both supernatants and cells, which was consistent mice heart with RT-qPCR results (Figure 8D,E).

### 3.11. Shikonin Inhibited Sea Gene Transcription and Expression

**Figure 9:**
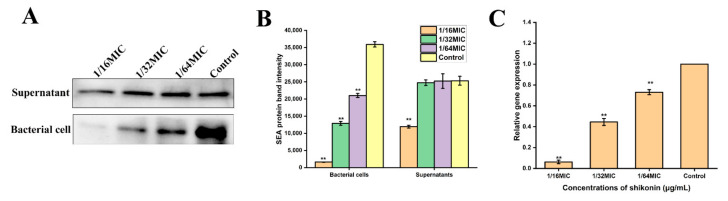
Non-inhibitory concentration of shikonin inhibited *S. aureus sea* gene expression. (**A**,**B**) Western Blot and densitometry detected the protein expression of SEA; (**C**) RT-qPCR detected the gene transcription of *sea*. ** *p* < 0.01.

When the strain ATCC29213 was treated with a non-inhibitory concentration of shikonin, the Western blot results show that the expression of SEA protein was significantly inhibited in the bacterial cells (*p* < 0.01) (Figure 9A,B). At the same time, the transcription of *sea* gene was also inhibited (Figure 9C).

### 3.12. The Thermal Stability and the Safety of Shikonin

After shikonin was treated at 100 °C for 1 min, 3 min, 5 min, even if the treatment temperature was increased to 121 °C, its MIC and MBC against *S. aureus* (ATCC 29213) were still 35 μg/mL, indicating that shikonin has good thermal stability (Table 3).

**Figure 10:**
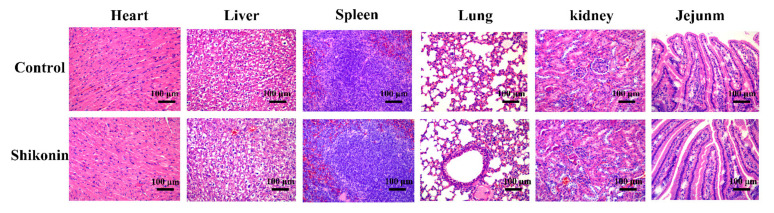
The major organs slice morphology (heart, liver, spleen, lung, kidney and small intestine) after 30 d of treatment with 800 mg/kg shikonin and PBS. The zoom ratio is 12.6%, and the magnification is 200×.

As shown in Figure 10, after the mice were treated with shikonin at 800 mg/kg once per day for 30 days, there was no obvious damage to the mice heart, liver, spleen, lung, kidney, and small intestine.

### 3.13. Application of Shikonin in Food Model

**Figure 11:**
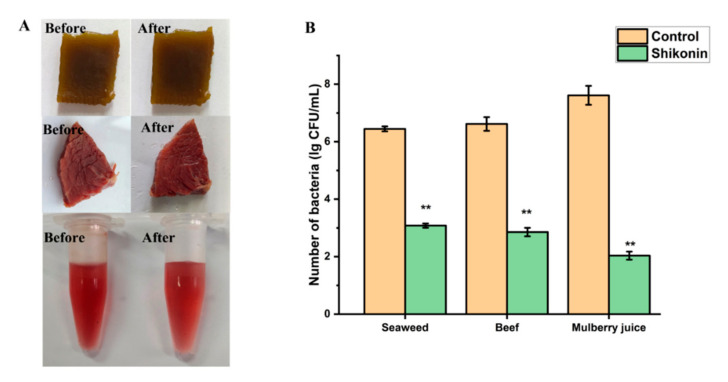
The application of shikonin in food. (**A**) The sensory morphology of food before and after shikonin treatment; (**B**) Shikonin inhibits the proliferation of *S. aureus* in food. ** *p* < 0.01.

In order to prove that shikonin can not only be used as a coloring agent in foods, but also has the potential to avoid *S. aureus* contamination, we chose three types of foods: sauced beef, seaweed, and mulberry juice. As shown in Figure 11B, the number of colonies in the untreated seaweed group was 6.45 log CFU/mL, and the number of colonies dropped to 3.08 log CFU/mL after shikonin pretreatment (*p* < 0.01). In the beef food model, the bacterial colony amount due to shikonin pretreatment decreased from 6.62 log CFU/mL to 2.86 log CFU/mL (*p* < 0.01). When shikonin was applied to mulberry juice, the antibacterial effect was the best. Compared with the untreated group, the number of colonies in the shikonin pretreatment group decreased by 5.58 log CFU/mL (*p* < 0.01). Moreover, through sensory evaluation, it was found that the addition of shikonin had no significant effect on the appearance of the food (Figure 11A).

## 4. Discussion

*S. aureus* food poisoning is a common foodborne disease caused by the ingestion of its enterotoxins [27]. Among the SEs, SEA is the most common toxin that causes food poisoning [1,3]. In addition to SEs, *S. aureus* also produces a variety of exotoxins, such as α-hemolysin, which is a pore-forming protein that has hemolytic, cytolytic and skin necrosis activities and contributes to the pathogenicity of *S. aureus* [28]. Likewise, the formation of biofilms brings great challenges to the elimination of *S. aureus*. Shikonin is a natural naphthoquinone compound extracted from comfrey root, and some current clinical animal experiments have proven that it has anti-inflammatory, anti-tumor and extensive antibacterial activities [29,30,31]. However, its antibacterial effect on foodborne *S. aureus* is rarely studied. Therefore, the purpose of this study was to evaluate the antibacterial activity of shikonin against foodborne *S. aureus* and its mechanism, and to broaden the biological functions of shikonin in food.

Many natural extracts have antibacterial activity against *S. aureus*. For example, the MICs of punicalagin, chlorogenic acid, resveratrol against *S. aureus* are 0.25 mg/mL, 0.256 mg/mL, 512 μg/mL, separately [32,33,34]. The above natural extracts reduced the virulence gene expression of *S. aureus* [33,34] and inhibited the biofilm formation of *S. aureus* [32]. In this study, the MIC of shikonin against *S. aureus* was 35–70 μg/mL. Shikonin also significantly reduced *S. aureus* virulence gene expression and inhibited its biofilm formation. Compared to the above natural extracts, shikonin has a better antibacterial activity against *S. aureus*. The reason may be related to the chemical structure of shikonin. Some previous studies have shown that quinone has certain antibacterial activity, and shikonin is a naphthoquinone compound, therefore it has an excellent antibacterial activity [35].

The potential difference between the two sides of the cell membrane in the resting state of the cell is defined as the membrane potential. Normal membrane potential is necessary for cell survival [36,37]. The membrane potential changes after receiving external stimuli: one is the increase in membrane potential, which means depolarization; the other is the decrease in membrane potential, which means hyperpolarization. The essence of depolarization is the flow of potassium ions from the cell to the extracellular matrix, and this abnormal flow of potassium ions is caused by cell membrane damage [38]. In this study, shikonin caused the depolarization of *S. aureus* cell membrane and a large amount of the fluorescent dye trimethoxazole DiBAC_4_(3) into *S. aureus* cells. Similarly, Wang et al. (2018) demonstrated that HJH-1 depolarized the cell membrane of *Escherichia coli* (*E. coli*) [20], which is a cationic peptide derived from the hemoglobin α subunit of rabbit red blood cells. Lee et al. (2016) also reported that (−)-Nortrachelogenin caused a large amount of the fluorescent dye trimethoxazole DiBAC_4_(3) into the *E. coli O157:H7* cells, which indicated that (−)-Nortrachelogenin caused the depolarization of the *E. coli* cell membrane. In addition, many people have reported that some natural products cause the hyperplasia of foodborne pathogens [38]. For example, coenzyme Q_0_ caused the hyperplasia of the *Cronobacter sakazakii* cell membrane, which led to cell membrane dysfunction [18]. Similarly, ferulic acid also caused the hyperplasia of *Cronobacter sakazakii* cell membrane and exhibit antibacterial activity against *Cronobacter sakazakii* [39]. It can be seen from the above that whether natural extracts cause depolarization or hyperplasia, this damages the integrity of the bacterial cell membrane.

The instability of the plasma membrane or the leakage of ions could affect the membrane-related energy conversion system. As an energy molecule in bacteria, ATP participates in various physiological processes. Many previous reports indicated that natural antibacterial substances caused the decrease of the intracellular ATP concentration or the increase of extracellular ATP concentration to exhibit antibacterial effects [18]. For example, Song et al. (2020) confirmed that the significant decrease of the intracellular ATP concentration of *S. aureus* caused by carboxymethylated β-glucan (CMG) [40]. Bajpai et al. (2013b) reported that the significant increase of extracellular ATP concentration of *E. coli O157*:*H7* and *B. cereus* was caused by eleutherococcus senticosus (ESEO), indicating that the cell membrane damage of the bacteria was caused by ESEO to exhibit antibacterial activity of ESEO against foodborne pathogens [41]. In this study, the intracellular ATP concentration of *S. aureus* was significantly reduced by shikonin. Combined with the above studies, they confirmed that the intracellular or extracellular ATP concentration can reflect the integrity of the cell membrane. However, the rapid hydrolysis of ATP should be considered. When the bacterial cell membrane was damaged, the intracellular and extracellular ATP concentration may decrease at the same time, resulting in inaccurate detection results. Therefore, rapid detection of ATP is required [42].

The cell membrane can separate the cell interior from the extracellular environment, while it maintains the material exchange function between the cell and the external environment. Once the cell membrane is damaged, it will cause the leakage of cell contents and even cell death [43]. In this study, SYTO 9 combined with PI double staining was used to quantify the damage of shikonin treatment to the cell membrane of *S. aureus*. Among them, SYTO 9 forms strong green fluorescence when it binds to DNA of both life and dead cells, while PI mainly binds to the nucleic acid of dead cells with damaged cell membranes to form red fluorescence. When two dyes exist at the same time, PI exhibits a stronger nucleic acid binding ability than SYTO 9, making SYTO 9 unable to bind to DNA [44]. In this study, the ratio of PI-labeled *S. aureus* cells in the shikonin treatment group increased more significantly than that in the control group, indicating that shikonin has a significant effect on the permeability of the cell membrane of *S. aureus*. Similarly, the study by Lacombe et al. (2012) showed that the treatment of *E. coli O157:H7* with anthocyanins significantly increased the permeability of the cell membrane, thereby exhibiting antibacterial activity [45]. Jia et al. (2019) reported that almost all cells were stained red by PI when *S. aureus* was treated with the photosensitizer S-Porphin sodium (S-PS), while a few cells were stained by SYTO 9, which indicated that S-PS exhibits antibacterial activity by damaging the cell membrane of *S. aureus* [44]. In this study the morphology of *S. aureus* was also observed by scanning electron microscopy, and the results showed that shikonin did not cause significant cell disintegration, which indicated that shikonin exert antibacterial activity might by binding to target molecules on the cell membrane of the bacteria [46]. In contrast, the study by Xu et al. (2017) showed that punicalagin severely damages the morphology of *S. aureus*, which is an important factor in inhibiting *S. aureus* [32].

The formation of biofilms has been extensively identified as an important factor in the continued existence of bacterial contamination of food [47,48]. In addition, the food production environment provides favorable conditions for the formation of biofilms, which brings greater challenges to the removal of pathogenic bacteria. Without exception, biofilm is one of the key virulence factors of *S. aureus*. In this study, it was found that the non-inhibitory concentration of shikonin significantly inhibited the formation of biofilm. Research by Liu et al. (2020) also reported similar results [49]. They reported that the formation of *Listeria monocytogenes* biofilm was significantly inhibited under shikonin treatment. Previous studies have reported that the transcription of *sarA* and *icaA* has a positive regulatory effect on the formation of biofilms [50,51]. Liu et al. (2020) confirmed that fusidic acid at non-inhibitory concentrations inhibits the biofilm biomass of *S. aureus* by inhibiting the transcription of *icaA* and *sarA* [49]. However, in this experiment, we found that shikonin inhibits the formation of biofilms by inhibiting the transcription of *sarA* and *cidA*, rather than inhibiting *icaA* and *agrA*. These results indicate that different natural products inhibit the biofilm formation of *S. aureus* by regulating the expression of different genes.

In addition to its toxicity, toxins secreted by *S. aureus* are a key pathogenic factor [52,53]. SEs and Hla are two main pathogenic factors for human [49], but natural extracts can directly inhibit the expression of *S. aureus* virulence factors at non-inhibitory concentrations [54]. For example, Duan et al. (2018) proved that the non-inhibitory concentration of resveratrol inhibits the expression of *S. aureus* α-hemolysin [34]. Research by Liu et al. (2020) showed that non-inhibitory concentrations of fusidic acid inhibited the expression of α-hemolysin by inhibiting the transcription of *saeRS* [49]. Peppermint oil inhibited the hemolytic activity of *S. aureus* ATCC 29,213 and inhibited the transcription and expression of *hla*, *sea*, and *seb* [55]. The results of this study are consistent with the above studies: the non-inhibitory concentration of shikonin significantly inhibited the hemolytic activity of *S. aureus* and inhibited the transcription and expression of *sea* and *hla*.

According to GB 2760-2014 “National Food Safety Standard Food Additives Use Standard” [56], alkannin, the isomer of shikonin, is mainly used as a coloring agent in beverages, biscuits, fruit and vegetable juices, flavored beverages, fruit wine, pastries, and canned spicy meat and poultry. It has confirmed that alkannin is safe for usage in food. This study confirms that shikonin has good thermal stability, indicating that the antibacterial activity of shikonin remained after high-temperature treatment during the canning process. In addition, shikonin added to food and can effectively inhibit and kill *S. aureus* in this study. Shikonin and its isomer alkannin have been used as colorant anda pigment antioxidant activities in foods in Europe and China [57,58]. In this study, the dosage of shikonin (0.0035% *w/w*) as a natural preservative to kill *S. aureus* was far lower than the dosage of shikonin (each 0.02% *w/w*) added to corn oil, olive oil and sunflower oil as an antioxidant [7]. At the same time, the dosage of shikonin used in this study is also far lower than the usage amount (0.1–1 mg/g) of the isomer alkannin as a colorant specified by the Chinese national standard. In this study, animal in vivo experiments showed that even continuous with high concentration intragastric gavage of shikonin with MIC concentration did not cause obvious damage to the ileum, heart, liver, spleen, lung, and kidney of mice. Therefore, our results in animal models indicate that shikonin may be safe to use in vivo. This is consistent with previous studies that demonstrated that shikonin derivatives may be safe to use in vivo in animal models [9,10]. In general, this shows that the dose of shikonin we use as a natural preservative in food is safe.

## 5. Conclusions

In conclusion, our research has proved that shikonin exhibits stronger antibacterial activity against *S. aureus* compared with most natural extracts. Shikonin destroyed the integrity of the cell membrane of *S. aureus*, depolarized the cell membrane, reduced the intracellular ATP concentration, changed cell morphology, and inhibited the transcription and expression of its virulence genes, thereby showing strong antibacterial activity. Because shikonin has a strong antibacterial activity against *S. aureus*, it can be used in food production and processing environments to effectively control *S. aureus*. However, more studies need to be carried out in the future to determine the toxicity and safety of shikonin as a natural food preservative against *S. aureus*.

## Figures and Tables

**Table 1:** Primers for RT-qPCR.

Gene	Primer Sequence (5′-3′)
*icaA*-F	GATACTGATATGATTACCGAAGAT
*icaA*-R	GAACCAACATCCAACACAT
*cidA*-F	ATTCATAAGCGTCTACACCTT
*cidA*-R	TTCTTCATACCGTCAGTTGT
*agrA*-F	TGAAATTCGTAAGCATGACCC
*agrA*-R	CATCGCTGCAACTTTGTAGAC
*sarA*-F	TGTTTGCTTCAGTGATTCGTTTA
*sarA*-R	AACCACAAGTTGTTAAAGCAGTTA
16SrRNA-F	CGTGCTACAATGGACAATACAAA
16SrRNA-R	ATCTACGATTACTAGCGATTCCA
*hla*-F	TTGGTGCAAATGTTTC
*hla*-R	TCACTTTCCAGCCTACT
*sea*-F	ATGGTGCTTATTATGGTTATC
*sea*-R	CGTTTCCAAAGGTACTGTATT

**Table 2:** MIC and MBC of Shikonin on 9 Strains of *Staphylococcus aureus*.

Strain	MIC (μg/mL)	MBC (μg/mL)	Original Source of Strain
ATCC29213	35	35	American Type Culture Collection
ATCC25923	35	35	American Type Culture Collection
A48	35	35	Patient anal swab
A86	35	35	Patient anal swab
13#	70	70	Grilled Chicken
124	70	70	Patient vomit
265	70	70	Patient vomit
S2	70	70	Patient vomit
S4	70	70	Patient vomit

**Table 3:** MIC and MBC of Shikonin against *Staphylococcus aureus* (ATCC 29213) strains after heat treatment.

Processing Conditions		MIC (μg/mL)	MBC (μg/mL)
Temperature (°C)	Time (Min)		
100	1	35	35
100	3	35	35
100	5	35	35
121	0.5	35	35

## Supplementary Materials

The following are available online at https://www.mdpi.com/article/10.3390/foods10122954/s1. Figure S1: The chemical structure of shikonin; Figure S2: The linear relationship between ATP concentration and luminescence; Figure S3: The linear relationship between the percentage of viable cells and the intensity of green fluorescence; Figure S4: RT-qPCR quantitative gene transcription. (A) The gene transcription of *icaA* of strain ATCC29213 treated with shikonin; (B) The gene transcription of *agrA* of strain ATCC29213 treated with shikonin. ** *p* < 0.01, * *p* < 0.05.
