# Supplementary material for: Control of Foodborne Staphylococcus aureus by Shikonin, a Natural Extract
Source: Foods. 2021 Dec 1;10(12):2954. doi: 10.3390/foods10122954 (PMC8700560; doi:10.3390/foods10122954)
Supplement: Supplementary file 1 [file foods-10-02954-s001.zip › foods-1384837-supplementary.pdf]

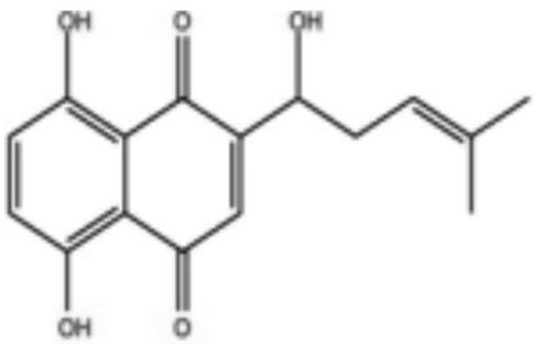

Figure S1. The chemical structure of shikonin.

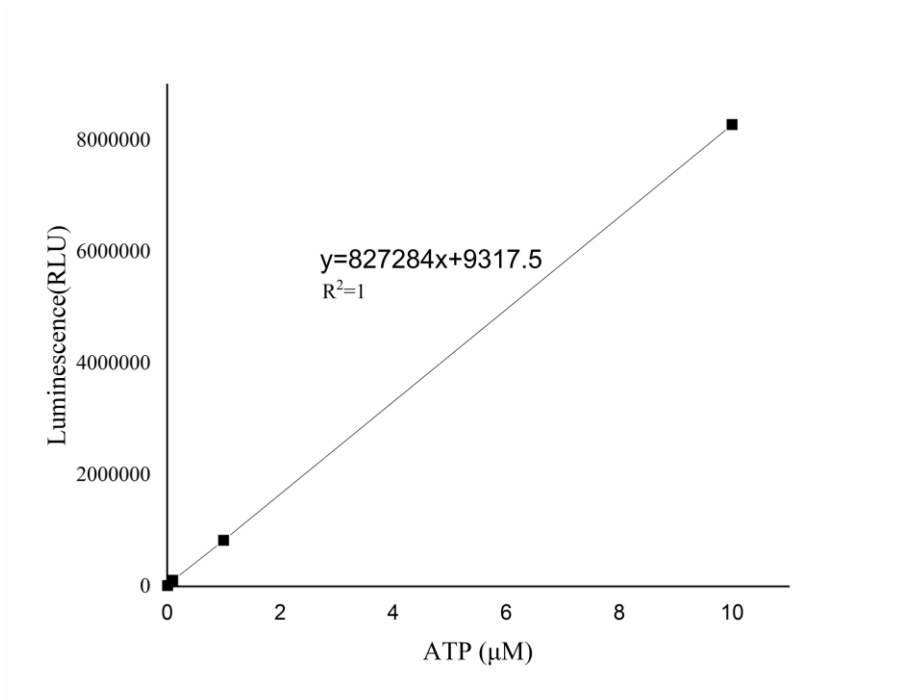

Figure S2. The linear relationship between ATP concentration and luminescence.

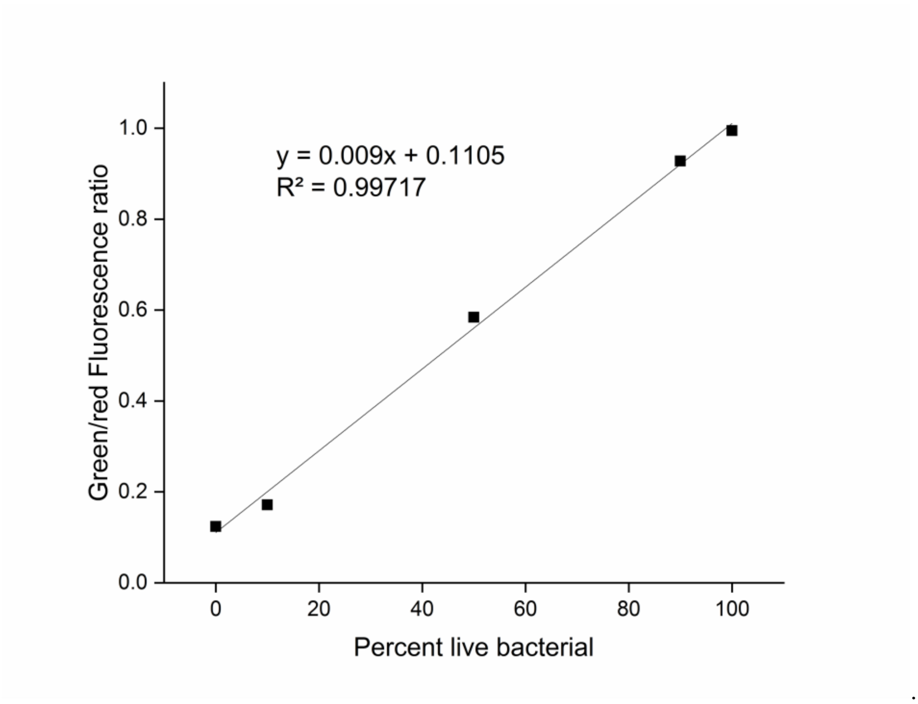

Figure S3. The linear relationship between the percentage of viable cells and the intensity of green fluorescence.

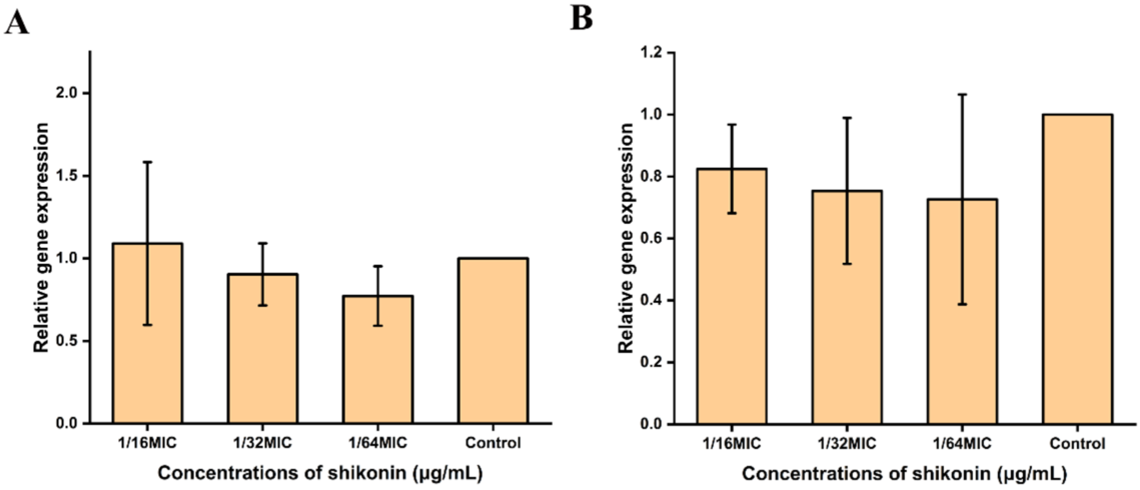

Figure S4. RT-qPCR quantitative gene transcription. (**A**) The gene transcription of *icaA* of strain ATCC29213 treated with shikonin; (**B**) The gene transcription of *agrA* of strain ATCC29213 treated with shikonin. $^{**}P < 0.01$, $^{*}P < 0.05$.
